# Two New Aluminoborates with 3D Porous-Layered Frameworks

**DOI:** 10.3390/molecules28114387

**Published:** 2023-05-27

**Authors:** Chen Wang, Juan Chen, Chong-An Chen, Zhen-Wen Wang, Guo-Yu Yang

**Affiliations:** MOE Key Laboratory of Cluster Science, School of Chemistry and Chemical Engineering, Beijing Institute of Technology, Beijing 100081, Chinacca@bit.edu.cn (C.-A.C.); wzw@bit.edu.cn (Z.-W.W.)

**Keywords:** aluminoborates, oxoboron cluster, 3D porous layers, solvothermal syntheses

## Abstract

Two new aluminoborates, NaKCs[AlB_7_O_13_(OH)]·H_2_O (**1**) and K_4_Na_5_[AlB_7_O_13_(OH)]_3_·5H_2_O (**2**), have been hydro(solvo)thermally made with mixed alkali metal cationic templates. Both **1** and **2** crystallize in the monoclinic space group *P*2_1_/*n* and contain similar units of [B_7_O_13_(OH)]^6−^ cluster and AlO_4_ tetrahedra. The [B_7_O_13_(OH)]^6−^ cluster is composed of three classical B_3_O_3_ rings by vertex sharing, of which two of them connect with AlO_4_ tetrahedra to constitute monolayers, and one provides an O atom as a bridging unit to link two oppositely orientated monolayers by Al-O bonds to form 3D porous-layered frameworks with 8-MR channels. UV-Vis diffuse reflectance spectra indicate that both **1** and **2** exhibit short deep-UV cutoff edges below 190 nm, revealing that they have potential applications in deep-UV regions.

## 1. Introduction

Crystalline borates send out an enchanting charm in the sciences because of their multifarious structures and widespread applications in microporous and nonlinear optical (NLO) materials [1,2,3,4,5]. In 1975, the NLO properties of KB_5_O_8_·4H_2_O [6] were studied by C F. Dewey et al. for the first time, pointing out the new research direction for the structure and properties of borates. Subsequently, high-temperature solid-state reactions and the boric flux method became the main methods of synthesizing borates [7,8]. Until 2004, Yang’s group applied the hydro(solvo)thermal method for the borate system and gradually introduced inorganic cations, organic amines, transition metal complexes, or chiral metal complexes as structure directing agents (SDAs) [9]. It is significant that the SDAs play an important role in the formation of structure by host–guest symmetry and charge matching [10], which effectively regulate the inorganic skeleton and successfully acquire abundant borates with novel open frameworks. In recent years, researchers have paid more attention to the alkali and alkaline earth metal borates [11] because of their better chemical stabilities, higher transmittances, greater damage thresholds, and almost no absorption properties of ultraviolet (UV) light [12], such as the well-known NLO materials: *β*-BaB_2_O_4_ (BBO) [13], CsLiB_6_O_10_ (CLBO) [14], and LiB_3_O_5_ (LBO) [15]. These research achievements have enormously inspired scientists’ enthusiasm and curiosity for pursuing newer borates.

In terms of structure, boron atoms typically adopt three or four coordination geometries with oxygen atoms to form BO_3_ triangles or BO_4_ tetrahedra. The combination of these two units via corner- or edge-sharing generates various oxoboron clusters, which can further polymerize through H-bonds and covalent bonds to constitute 1D chains, 2D layers, and 3D frameworks [16,17,18,19,20]. Moreover, in order to expand the structural diversity of borates, Al^3+^ was introduced into the borates’ framework [21]. It is worth noting that Al is in the same group as boron but has more plentiful coordination modes, such as the AlO_4_ tetrahedron, AlO_5_ tetragonal pyramid, and AlO_6_ octahedron [22,23,24]. The developments of aluminoborates (ABOs) were slow since Al was firstly led into the borates system by Lehmann and Teske in 1973 [25], mainly because the single crystal structures of limited ABOs were difficult to be determined [26]. However, this difficulty was solved in 2009. Our group put forward the use of Al(i-PrO)_3_ to replace the traditional inorganic Al sources under hydro(solvo)thermal conditions [27]. Except for its better solubility in organic solvents, the synergism between chiral AlO_4_ tetrahedra formed in hydrolysis progress and acentric structures [28]. By guiding with different types of SDAs, numerous ABOs have been reported [29,30,31,32].

Herein, two novel 3D porous layered ABOs, NaKCs[AlB_7_O_13_(OH)]·H_2_O (**1**) and K_4_Na_5_[AlB_7_O_13_(OH)]_3_·5H_2_O (**2**), were solvothermally made. The 3D porous-layered frameworks of **1** and **2** were both built by the alternation of [B_7_O_13_(OH)]^6−^ clusters and AlO_4_ units. The [B_7_O_13_(OH)]^6−^ cluster was composed of three classical B_3_O_3_ rings, of which two of them constructed monolayers with AlO_4_ tetrahedra, and adjacent layers were connected through bridging O atoms provided by another B_3_O_3_ ring. The evident difference between **1** and **2** was that the asymmetric unit of **2** contained three crystallographically independent [AlB_7_O_13_(OH)]^3−^, and they were linked, in turn, along the *b*-axis. Different cationic diameters also resulted in the diverse curvature of the porous layers. The structure, comparison, and synthesis of the above two compounds will be discussed in detail in the following sections.

## 2. Results and Discussion

### 2.1. Synthesis Procedure

Compounds **1** and **2** both adopted two kinds of boron sources as reactants, and the products could not be obtained without any one, which were confirmed through experiments. Furthermore, **1** used H_3_BO_3_ and Na_2_B_4_O_7_·10H_2_O, while **2** used H_3_BO_3_ and NaBO_2_·4H_2_O. Wherein, H_3_BO_3_, through self-polymerization, could build various oxoboron clusters. Meanwhile, polyborate (Na_2_B_4_O_7_·10H_2_O, NaBO_2_·4H_2_O) could not only enhance the pH of whole system but also may further recombine new oxoboron clusters through the degradation of polyanions. In addition, the reaction temperature and pH were also major factors affecting the reaction, and higher temperatures and pH levels were more conducive to improving the polymerization of the oxoboron clusters (Temperature: 210 °C for **1** and 230 °C for **2**; pH: 7 for **1** and 9 for **2**). The possible chemical equations during the reaction are given below, respectively.

3H_3_BO_3_ + [B_4_O_7_]^2−^ + 4OH^−^ + Al^3+^ = [AlB_7_O_13_(OH)]^3−^ + 6H_2_O (1)

H_3_BO_3_ + 13[BO_2_]^−^ + 2Al^3+^ = 2[AlB_7_O_13_(OH)]^3−^ + OH^−^ (2)

### 2.2. Structure of ***1***

**Figure 1:**
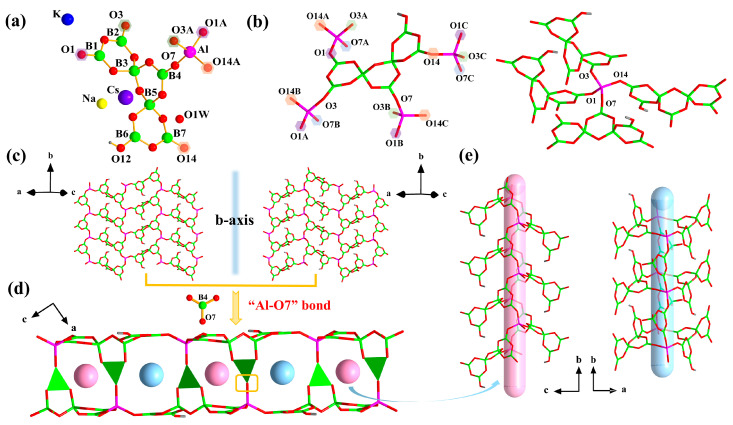
(**a**) Asymmetric unit of **1**; (**b**) Coordination environment of B_7_ cluster and AlO_4_ tetrahedra in **1**; (**c**) Two orientated monolayers in **1**; (**d**) The 3D porous-layered framework with two channels in **1**; (**e**) View of the two types of the 8-MR channels.

Single crystal X-ray analyses display that **1** crystalizes in the monoclinic space group *P*2_1_/*n*. The asymmetric unit of **1** contains one [B_7_O_13_(OH)]^6−^ cluster, one Na, one K, one Cs, and one water molecule (Figure 1a). The [B_7_O_13_(OH)]^6−^ cluster consists of three familiar B_3_O_3_ rings, in which five BO_3_ triangle units and two BO_4_ tetrahedral groups are connected by vertex sharing. The B-O distances in the range of 1.329 Å to 1.402 Å, and the O-B-O bond angles lie in the range of 114.9°–122.7° for the BO_3_ triangles. Meanwhile, the B-O distances range from 1.427 Å to 1.511 Å, and the O-B-O bond angles vary from 106.8° to 148.9° for BO_4_ tetrahedra.

Each [B_7_O_13_(OH)]^6−^ cluster was further connected to four AlO_4_ tetrahedral via O1, O3, O7, and O14 atoms, and vice versa (Figure 1b). The alternating connection of oxoboron clusters and AlO_4_ tetrahedra formed a 2D fluctuating monolayer with 13-MR windows, which were arranged in two orientations (Figure 1c). The windows consisted of Al-B2-B3-B5-B7-Al-B2-B1-Al-B7-B5-B3-B1(Figure S1a). Two monolayers exhibited axial symmetry along the *b*-axis and were linked by Al-O bonds, constituting a 3D double-layer structure with two unequal types of 8-MR channels, which possessed the same components but had different shapes. These two channels were delineated by Al-B1-B3-B4-Al-B2-B3-B4 (Figure S1b) and placed in an -ABAB- sequence in the *ac* plane (Figure 1d,e). 

**Figure 2:**
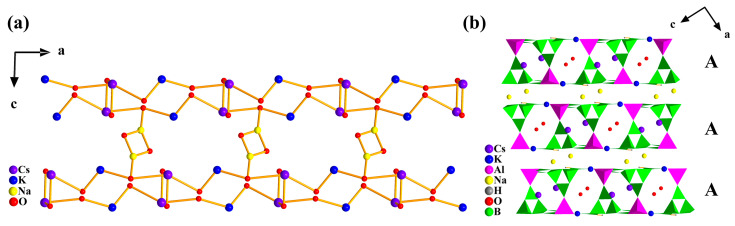
(**a**) The metal–oxygen net in **1**; (**b**) The complete dense 3D network in **1**.

As for the coordination of metal cations, K was surrounded by eight O atoms, Na was coordinated by seven O atoms, and Cs was bonded by eight O atoms (Figure S2). It should be noted that these metal atoms shared oxygen atoms to lead a three-dimensional metal–oxygen net similar to a ladder (Figure 2a). Specifically, Na atoms were located between adjacent porous layers, K atoms were situated on the wall of 8-MR channels, and Cs and O1W atoms were seated in the neighboring channels, respectively. In general, the porous layers stacked along the *ac* plane in an -AAA- sequence, composing the complete dense 3D network with cations (Figure 2b).

### 2.3. Structure of ***2***

**Figure 3:**
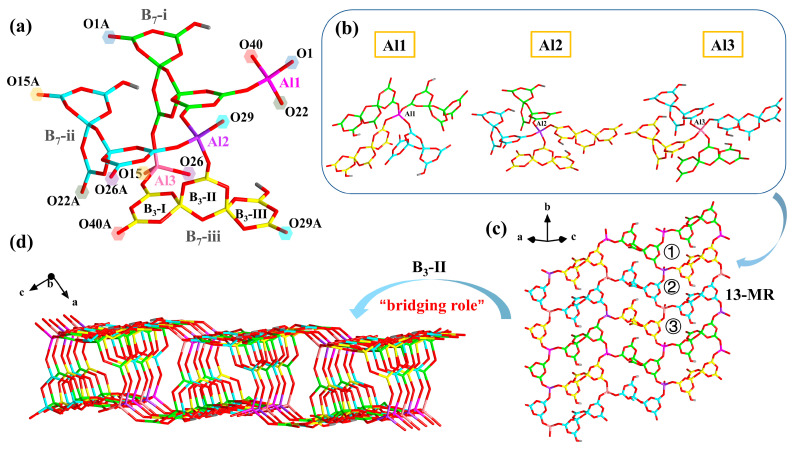
(**a**) Asymmetric unit of **2**; (**b**) Coordination environment of three different AlO_4_ tetrahedra in **2**; (**c**) The monolayer with three kinds of 13-MR rings in **2**; (**d**) The 3D framework in **2**.

Notably, **2** crystallized in the monoclinic space group *P*2_1_/*n*, and its asymmetric unit contained three crystallographically independent [AlB_7_O_13_(OH)]^3−^, four K atoms, five Na atoms, and five water molecules (Figure 3a). The [B_7_O_13_(OH)]^6−^ cluster was the same as it was in **1,** and the distances of the B-O bond varied from 1.339 Å to 1.521 Å.

**Figure 4:**
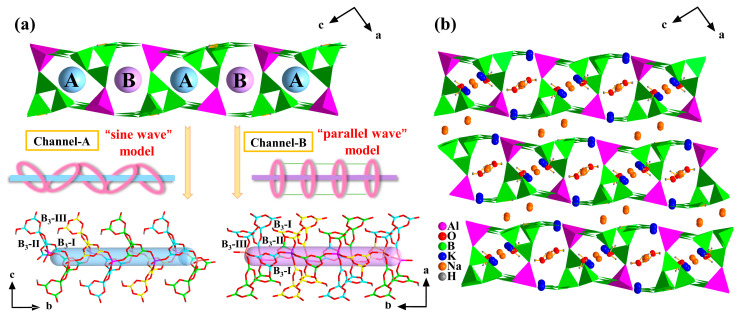
(**a**) View of two different types of channels in **2**; (**b**) The 3D porous-layered framework in **2**.

Each AlO_4_ tetrahedra connected with four neighboring {B_7_} clusters of three different types. The Al1 was bonded with two B_7_-i, one B_7_-ii, and one B_7_-iii. The Al2 was linked with two B_7_-iii, one B_7_-I, and one B_7_-ii, whereas Al3 was joined to two B_7_-ii, one B_7_-I, and one B_7_-iii, separately (Figure 3b). These three [AlB_7_O_13_(OH)]^3−^ clusters were connected, in turn, along the *b*-axis, constituting a monolayer with three kinds of 13-member rings (Figure 3c). The AlO_4_ tetrahedra and B_3_O_3_-II (B_3_-II) rings were interconnected by sharing O atoms, linking two adjacent single layers with opposite orientations into a 3D porous structure, and the porous layers were stacked in an -AAA- sequence in the *ac* plane (Figure 4b). From this point of view, the B_3_O_3_-I (B_3_-I) and B_3_O_3_-III (B_3_-III) connected with the AlO_4_ tetrahedra to constitute monolayers, while the B_3_-II as a bridging unit linked two oppositely orientated layers to the 3D frameworks (Figure 3d). 

Two kinds of channels existed, and each B_3_O_3_ ring played a different role (Figure 4a). Channel A was made of two AlO_4_ tetrahedra, two B_3_-I, and B_3_-II rings. The B_3_-I was responsible for bonding adjacent AlO_4_ tetrahedra in order to extend along the *b*-axis, while B_3_-II played an effect on linking the B_3_-I and AlO_4_ tetrahedra to form a closed window. The B_3_-III could be seen as a decoration hanging on the channel wall. However, the situation of channel B was diverse. It consisted of two AlO_4_ tetrahedra, two B_3_-II, and B_3_-III rings. The B_3_-II and B_3_-III only played a part in a closed window, while another B_3_-I linkedup the neighboring windows. From this perspective, channel B was composed of parallel windows, as channel A’s were linked end to end. In view of this, channel A could be seen as a “sine wave” model, while channel B could be regarded as a “parallel wave” model.

As for the metal cations, an Na atom was coordinated with seven O atoms, and the K atom was surrounded by five O atoms (Figure S3). It is worth noting that Na1 and the water molecules were filled in each channel, and Na2 was located in the interval between contiguous porous layers. Likewise, the K atoms had two locations. K1 was situated in channel A, and K2 was seated on the wall of channel B (Figure 4b). The metal–oxygen chain extended along *a*-axis and combined with the B-O network and the AlO_4_ tetrahedra, enhancing the stability of compound **2** (Figure S4).

### 2.4. Structure Comparison

To discuss in detail, compounds **1** and **2** exhibited a few similarities as well as distinctions. On the one hand, there were the same fundamental building blocks (FBBs) of both two, namely, [AlB_7_O_13_(OH)]^3−^, constituting the similar 3D porous-layered frameworks. On the other hand, the asymmetric unit of **2** contained three crystallographically independent [AlB_7_O_13_(OH)]^3−^, and they were connected, in turn, along the *b*-axis, being consistent with the cell parallel of **2**, being three times longer than that of **1**. Meanwhile, the cations were dissimilar to induce the various distortion of porous layers due to the different ionic radius. Furthermore, **1** was the approximately parallel layer, and **2** was the fluctuant layer, showing the distinct shapes for channels, whereas the aperture of **1** was even larger. Moreover, it was significant that the cations were in different positions of the two compounds: the K^+^ in **2** replaced the Cs^+^ in **1**, and a part of Na^+^ was filled in the channels of **2**, whereas they were only located in the interlayers in **1**. Additionally, there were more water molecules in **2**, situated in each channel, and the abundant hydrogen bonds made the whole structure more stable. 

To date, there have been limited 3D porous-layered ABOs reported on, such as [H_3_O]K_3.52_Na_3.48-_{Al_2_[B_7_O_13_(OH)][B_5_O_10_][B_3_O_5_]}[CO_3_] [33] (**3**), K_2_[Al_2_B_7_O_14_(OH)(en)_0.5_]·H_2_O [34] (**4**), and Ba_3_Al_2_[B_3_O_6_(OH)]_2_[B_4_O_7_(OH)_2_] [35] (**5**), with their respective characteristics. Firstly, the kind of window related to oxoboron clusters participated in the consistency of monolayers (Figure S5). There was one type of window in **1** and **5** because the monolayer was formed by single oxoboron cluster, whereas three types of windows in **2** and **3** existed, owing to three oxoboron clusters that all made contributions to the monolayers, the same state for **4**. Secondly, the aperture of the window was influenced by the oxoboron clusters’ sizes. There were larger windows in **1**, as its FBBs were composed of seven BO_3/4_ units. The same 13-MR window also occurred in **3**, but the [B_5_O_10_]^5−^ and [B_3_O_7_]^5−^ clusters were not enough to support such a large ring. Thus, a part of the 13-MRs were split into 8-MR and 10-MR. Thirdly, the bridging unit of the porous layers was different (Figure S6). The oxoboron clusters originating from monolayers providing the bridging units in compounds **1**–**3**, and the AlO_4_ tetrahedra were effective of this in **4**. However, in compound **5**, the individual [B_4_O_7_(OH)_2_]^4−^ cluster only played a part in connecting the adjacent monolayers. In terms of structure, there were unprotonated B_3_O_3_ rings perpendicular to the monolayers in **1**–**3**, which made their own could act as bridging units. However, in **4** and **5**, the terminal oxygens, extending outward, were all protonated. Thus, only other units could act as bridging units in these frameworks. Fourthly, the warping degree of the porous layers was diverse. The frameworks of **1**, **4**, and **5** were approximately parallel layers, possibly because the larger cationic radius made an effect on supporting the channels in Cs^+^ and Ba^2+^, while ethylenediamine molecules played this role in **4**. However, there was K^+^ or Na^+^ in **2** and **3**, making them show the fluctuant layers.

### 2.5. Powder XRD Patterns

The experimental PXRD patterns of **1** and **2** were consistent with the single crystal data’s simulated patterns, which illustrated that the samples were phase pure. The disagreement of the diffraction peak intensities between the experimental and simulated patterns were caused by the variations in the crystal orientations of the samples (Figure S7).

### 2.6. IR Spectra

It was homologous for **1** and **2** that the absorption bands and peaks were within 4000–500 cm^−1^ in the infrared spectra. Thus, only **1** was described in detail. The absorption peaks at 3440 cm^−1^ were the stretching vibrations of the -OH groups, while the peaks at 1624 cm^−1^ were the vibrations of H-O-H. The absorption bands ranging from 1445 to 1213 cm^−1^ were in accord with the asymmetric stretching of B-O in BO_3_ units, and the bands from 1095 to 990 cm^−1^ were attributed to the asymmetric stretching of the BO_4_ units. The peaks at 905 and 850 cm^−1^ were assigned to the symmetric stretching of BO_3_ and BO_4_, individually. The bands from 728 to 675 cm^−1^ belonged to the bending vibrations of these units. Moreover, the peaks in the range of 787 to 768 cm^−1^ corresponded with the stretching vibrations of the AlO_4_ groups (Figure S8). 

### 2.7. UV-Vis Absorption Spectra

**Figure 5:**
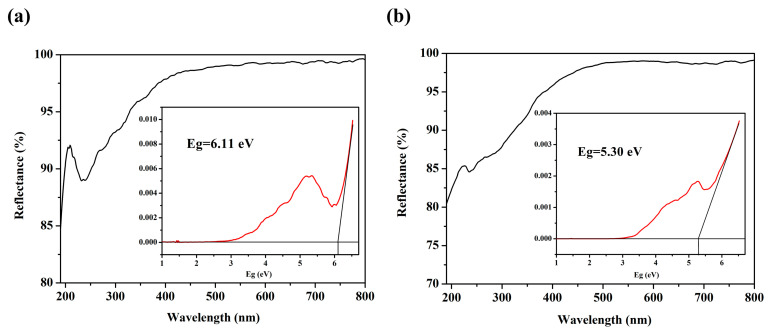
UV-Vis absorption spectra of **1** (**a**) and **2** (**b**).

As shown in Figure 5, the UV-Vis diffuse reflectance spectra that has been tested ranged from 190 to 800 nm. The Kubelka–Munk function *F*(*R*) = (1 − *R*)^2^/2*R* = *α/S* was used to calculate the absorption date (*α/S*), where *R* was the reflectance, *α* was the absorption coefficient, and *S* was the scattering coefficient. The band gaps of **1** and **2** were 6.11 eV and 5.30 eV, indicating that they were wide-band semiconductors. The UV cut-off edges of both **1** and **2** were below 190 nm, revealing that they had potential applications in ultraviolet regions.

### 2.8. Thermal Analysis

The thermal properties of compounds **1** and **2** were measured under the air atmosphere with a heating rate of 10°/min from 25 to 1000 °C. The 5.14% (Cal: 5.01%) weight losses from 125 °C to 463 °C in **1** and 8.97% (Cal: 8.70%) in the range of 102 °C to 441 °C in **2** were due to the removal of water molecules and the dehydration of -OH groups (Figure S9).

## 3. Materials and Methods

### 3.1. General Procedure

All chemical reagents were commercially available and used without further purification. Powder X-ray diffraction (PXRD) patterns were collected on a Bruker D8 Advance X-ray diffractometer with Cu K*α* radiation (*λ* = 1.54056 Å) in the angular range of 2*θ* scanning from 5–50° at room temperature. Infrared (IR) spectra were tested on a Nicolet iS10 instrument with wavenumbers ranging from 4000 to 40 cm^−1^. UV-Vis diffuse reflectance spectra were recorded in the range of 190–800 nm on a Shimadzu UV-3600 spectrometer. Thermogravimetric analyses were performed on a Mettler Toledo TGA/DSC 1100 analyzer from 25 to 1000 °C, with a heating rate of 10 °C h^−1^, under an air atmosphere.

### 3.2. Syntheses

#### 3.2.1. Syntheses of **1**

A mixture of H_3_BO_3_ (0.123 g, 2.0 mmol), K_2_B_4_O_7_·4H_2_O (0.159 g, 0.5 mmol), Na_2_B_4_O_7_·10H_2_O (0.193 g, 0.5 mmol), Cs_2_CO_3_ (0.187 g, 0.5 mmol), and Al(i-PrO)_3_ (0.206 g, 1.0 mmol) was added into a mixed solution of 3 mL ethanol and 2 mL distilled water. After continuous stirring for 2 h at room temperature, the resulting solution was sealed in a 25 mL Teflon-lined stainless-steel autoclave. Subsequently, it was heated in an oven at 210 °C for 5 days under an autogenous pressure. The colorless lamellar crystals were obtained after cooling down to room temperature and being washed with distilled water (Figure S10). 

#### 3.2.2. Syntheses of **2**

A mixture of H_3_BO_3_ (0.362 g, 6.0 mmol), NaBO_2_·4H_2_O (0.288 g, 2.0 mmol), K_2_CO_3_ (0.063 g, 0.5 mmol), and Al(i-PrO)_3_ (0.211 g, 1.0 mmol) was added into a mixed solution of 4 mL ethanol and 1 mL distilled water with constant stirring for 1h. Then, it was sealed in a 25 mL Teflon-lined stainless-steel autoclave and heated at 230 °C for 5 days. The colorless block crystals were obtained under the same procedures as **1**.

### 3.3. X-ray Crystallography

The single crystal X-ray diffraction data of **1** and **2** were tested and collected on a Gemini A Ultra CCD diffractometer with graphite monochromated Mo Kα (*λ* = 0.71073 Å) radiation in the *ω* scanning mode at room temperature. The structures were solved by direct methods and refined on *F*^2^ by the full-matrix least-squares method with the SHELX-2014 program package [36]. All non-hydrogen atoms in the compounds were refined with anisotropic displacement parameters. The hydrogen atoms were placed by geometrical calculations and fixed through structural refinement. Crystallographic data were deposited with the Cambridge Crystallographic Data Centre: CCDC 2256812 for **1** and CCDC 2256819 for **2**. Detailed crystallographic data of two compounds are listed in Table 1.

## 4. Conclusions

In summary, two new aluminoborates with mixed alkali metal cations were successfully obtained under hydrothermal conditions. Both **1** and **2** included the same fundamental building units, [B_7_O_13_(OH)]^6−^ clusters, and AlO_4_ tetrahedra, and the alternation of them made four connected networks with 8-MR channels and 13-MR windows along the *b*-axis, constituting the 3D porous-layered frameworks. The UV-Vis diffuse reflectance spectra indicated that both **1** and **2** exhibited the short deep-UV cutoff edges below 190 nm, and the bandgaps of them were 6.11 and 5.30 eV, revealing that they had potential applications in deep-UV regions. The successfully synthesis of the two above novel structures expanded the possibilities of ABOs structures and revealed the effect of metal cations on constructing frameworks. In the future, we will continue to explore the synthesis of distinctive ABOs with various alkali and alkaline earth metals.

## Figures and Tables

**Table 1:** Crystallographic data and structural refinements for **1**, **2**.

	1	2
Formula	NaKCsAlB_7_O_15_H_3_	K_4_Na_5_Al_3_B_21_O_47_H_13_
Molecular weight	540.66	1346.41
Crystal system	Monoclinic	Monoclinic
Space group	*P*2_1_/*n*	*P*2_1_/*n*
a/Å	11.3647 (16)	11.6261 (3)
b/Å	6.9730 (8)	20.9721 (6)
c/Å	17.6729 (19)	16.7820 (5)
*α*/°	90	90
*β*/°	91.880 (10)	93.646 (2)
*γ*/°	90	90
V/Å^3^	1399.8 (3)	4083.6 (2)
Z	4	4
Dc/g cm^−3^	2.556	2.183
μ/mm^−1^	3.118	0.699
*F*(000)	1016	2648
Goodness-of-fit on *F*^2^	1.069	1.079
R indices [*I* > 2*σ*(*I*)] ^1^	0.0484 (0.1046)	0.0471 (0.1519)
R indices (all data)	0.0751 (0.1216)	0.0526 (0.1563)

^1^ *R*_1_ = Σ||*F*_0_| − |*F*_c_||/Σ|*F*_0_|. w*R*_2_ = {Σw[(*F*_0_)^2^ − (*F*_c_)^2^]^2^/Σw[(*F*_0_)^2^]^2^}^1/2^.

## Data Availability

The raw data supporting the conclusions of this article will be made available by the authors without undue reservation.

## Supplementary Materials

The following supporting information can be downloaded at: https://www.mdpi.com/article/10.3390/molecules28114387/s1, Figure S1: Composition of 13-MR (a) and 8-MR (b) windows in **1**; Figure S2: The coordination of metal cations in **1**; Figure S3: The coordination of metal cations in **2**; Figure S4: Metal-oxygen chain in **2**; Figure S5: The 2D monolayers in **1** (a), **3** (b), **4** (c), and **5** (d), respectively; Figure S6: The porous-layered structures in **3** (a), **4** (b), and **5** (c), respectively; Figure S7: PXRD of **1** (a) and **2** (b); Figure S8: IR spectra of **1** (a) and **2** (b); Figure S9: TG-DSC curves of **1** (a) and **2** (b); Figure S10: The morphology of compounds **1** and **2**, respectively.
