# Supplementary material for: Two New Aluminoborates with 3D Porous-Layered Frameworks
Source: Molecules. 2023 May 27;28(11):4387. doi: 10.3390/molecules28114387 (PMC10254741; doi:10.3390/molecules28114387)
Supplement: Supplementary file 1 [file molecules-28-04387-s001.zip › molecules-2400753-supplementary.pdf]

molecules

Syntheses and structures of two new aluminoborates with 3D porous-layered frameworks

Chen Wang, Juan Chen, Chongan Chen, Zhenwen Wang and Guoyu Yang *

Figure S1. Composition of 13-MR (a) and 8-MR (b) windows in **1**.
Figure S2. The coordination of metal cations in **1**.
Figure S3. The coordination of metal cations in **2.**
Figure S4. Metal-oxygen chain in **2**.
Figure S5. The 2D monolayers in **1** (a), **3** (b), **4** (c) and **5** (d), respectively.
Figure S6. The porous-layered structures in **3** (a), **4** (b) and **5** (c), respectively.
Figure S7. PXRD of **1** (a) and **2** (b).
Figure S8. IR spectra of **1** (a) and **2** (b).
Figure S9. TG-DSC curves of **1** (a) and **2** (b).
Figure S10. The morphology of compounds **1** and **2**, respectively.

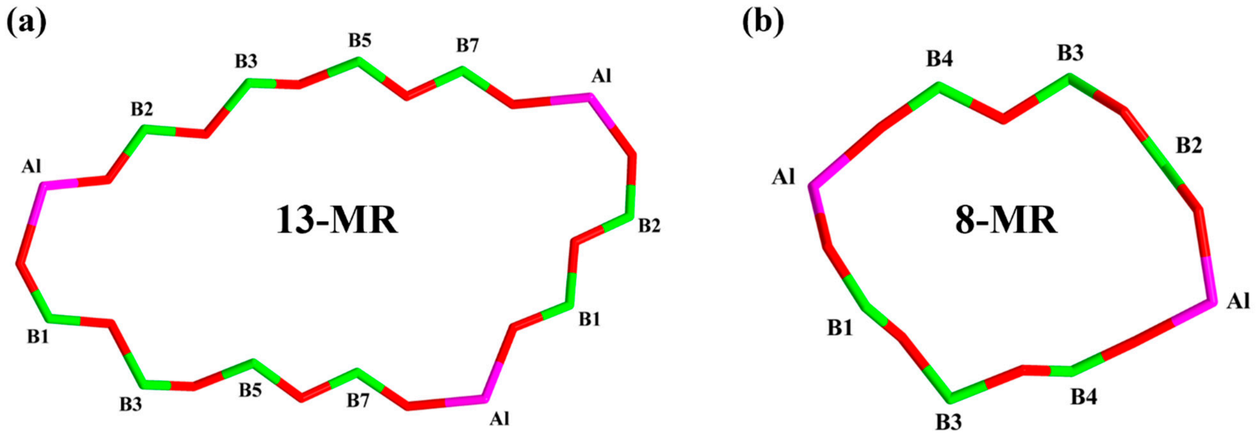

Figure S1. Composition of 13-MR (a) and 8-MR (b) windows in **1**.

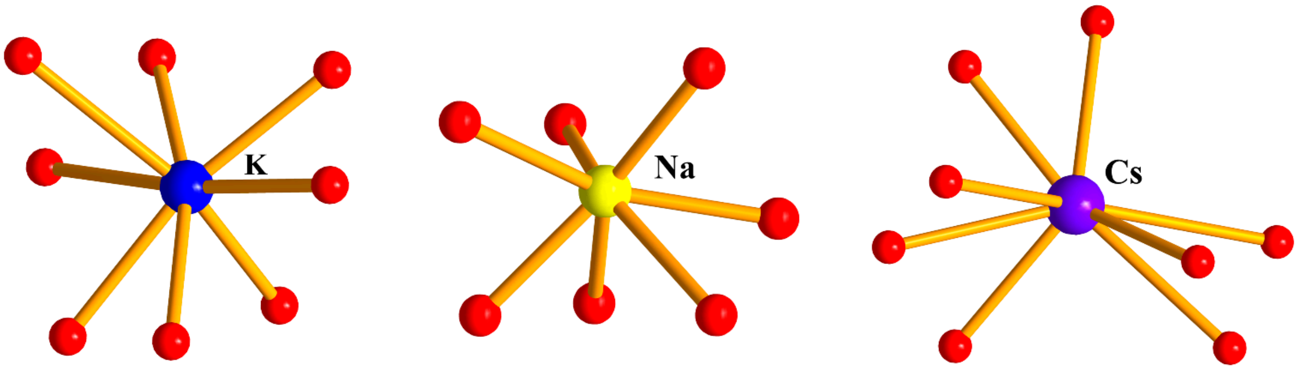

Figure S2. The coordination of metal cations in **1**.

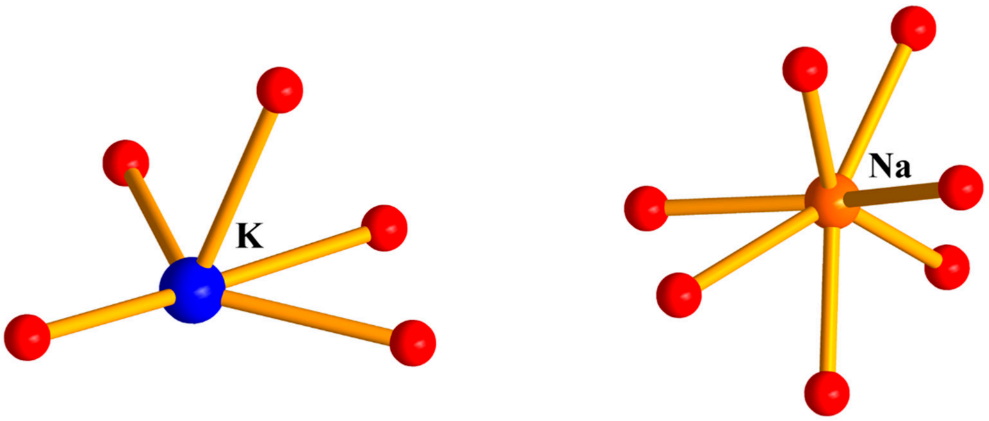

Figure S3. The coordination of metal cations in **2**.

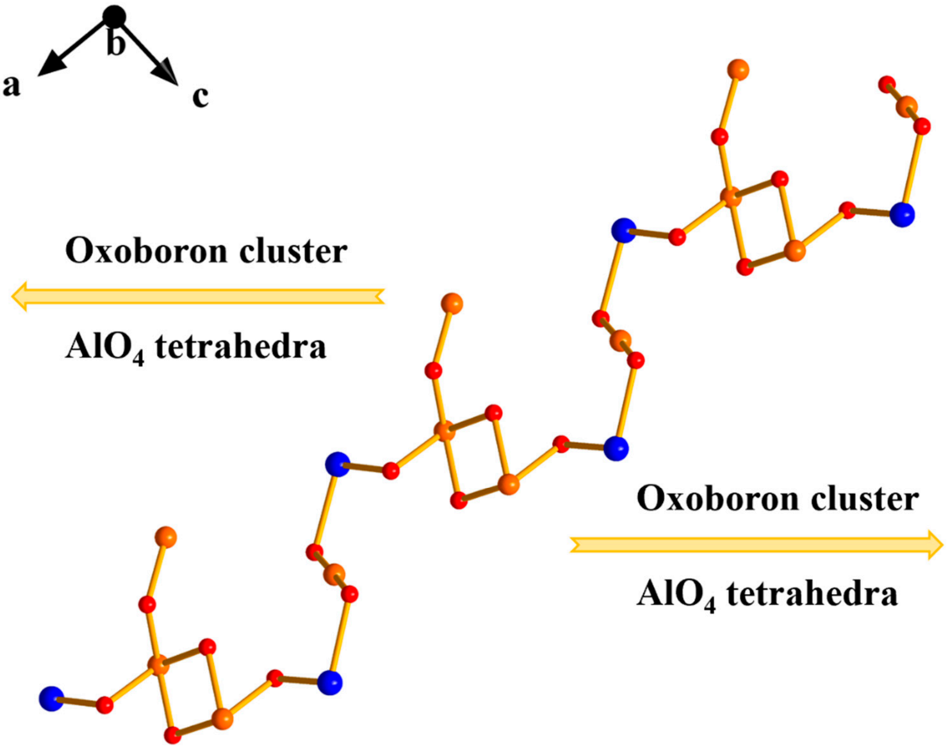

Figure S4. Metal-oxygen chain in **2**.

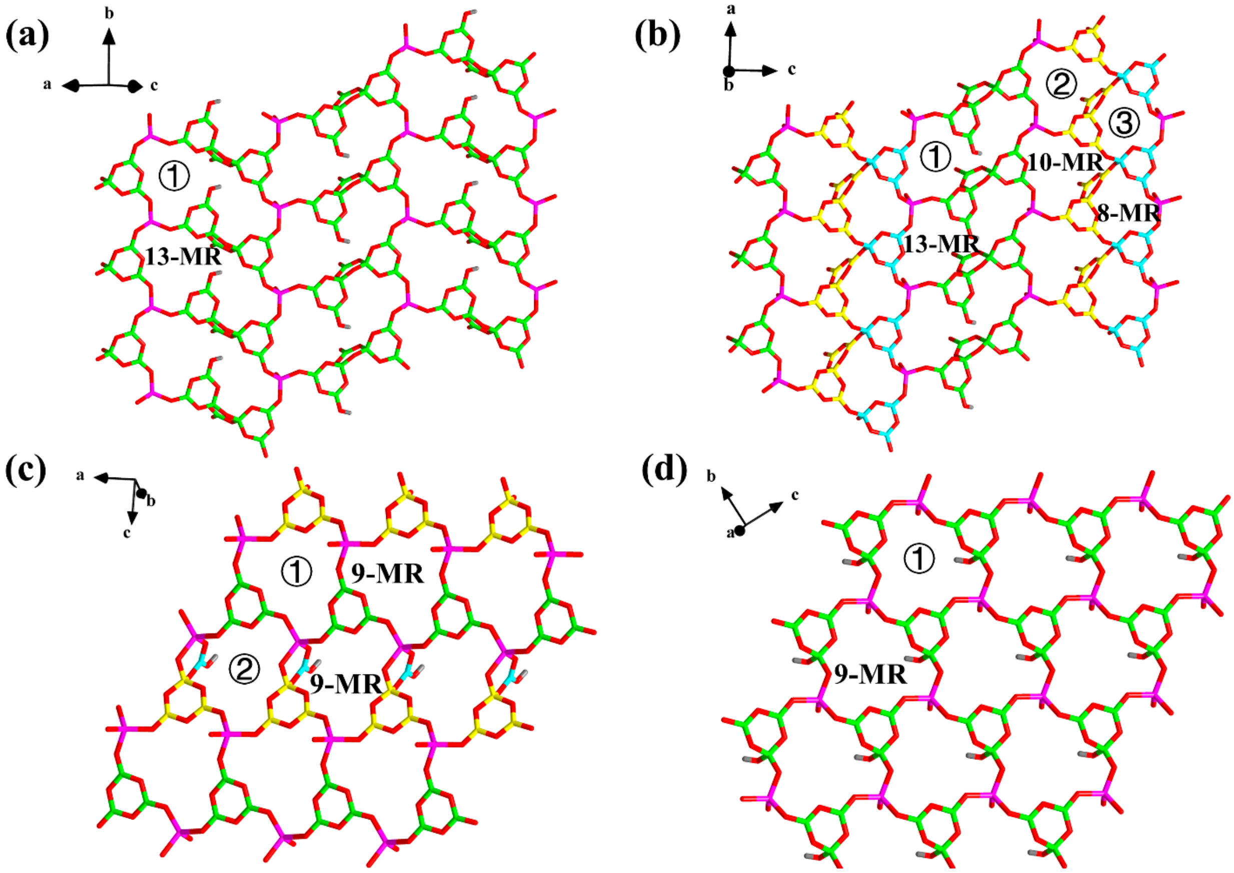

Figure S5. The 2D monolayers in **1** (a), **3** (b), **4** (c) and **5** (d), respectively.

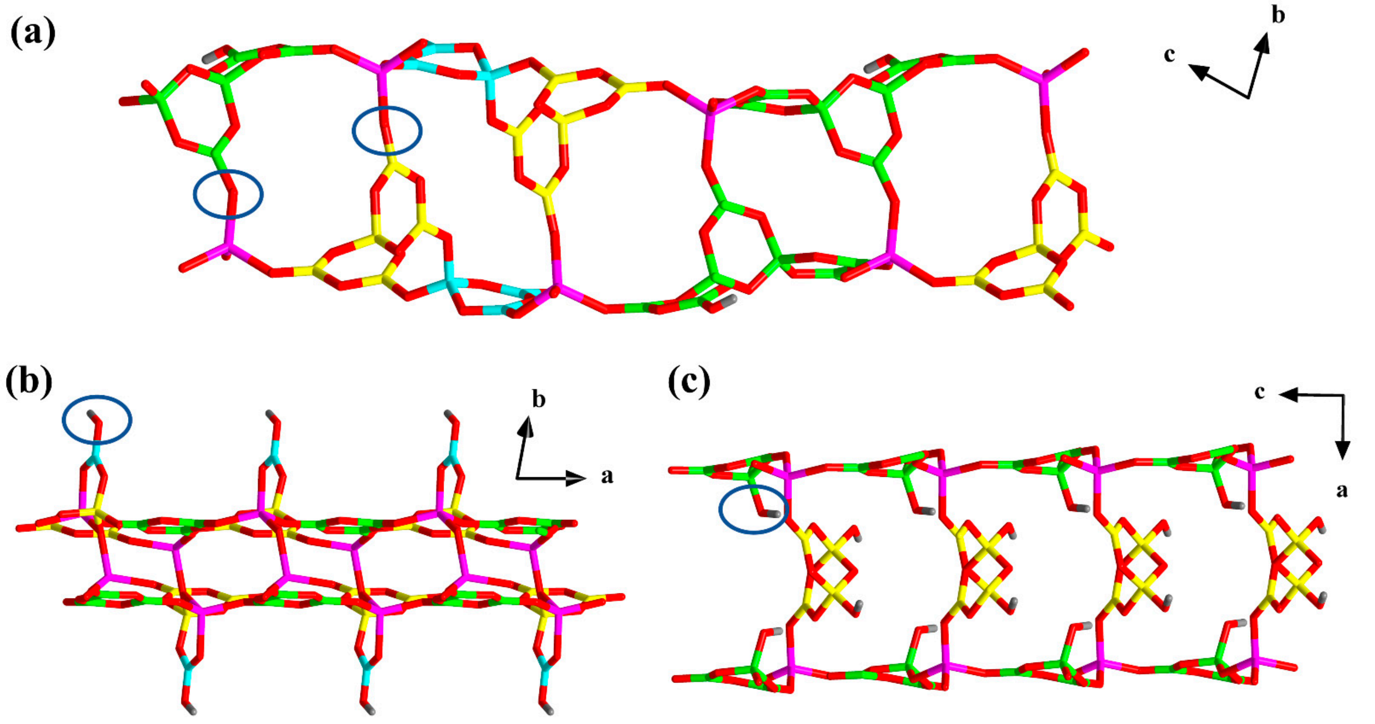

Figure S6. The porous-layered structures in **3** (a), **4** (b) and **5** (c), respectively.

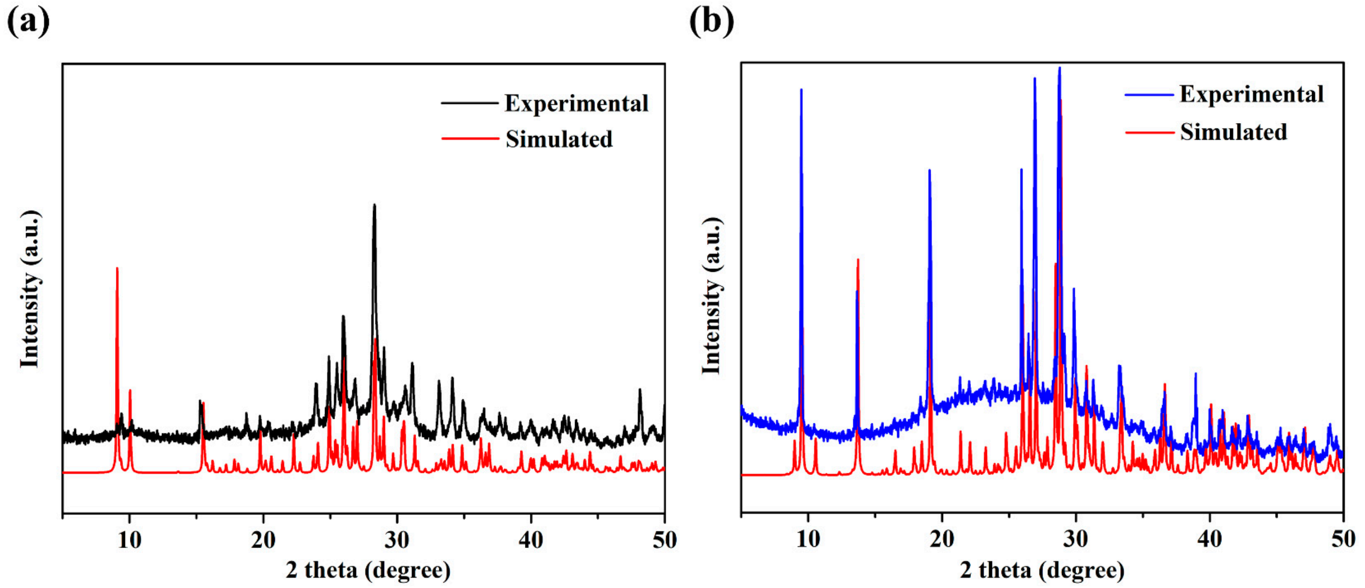

Figure S7. PXRD of **1** (a) and **2** (b).

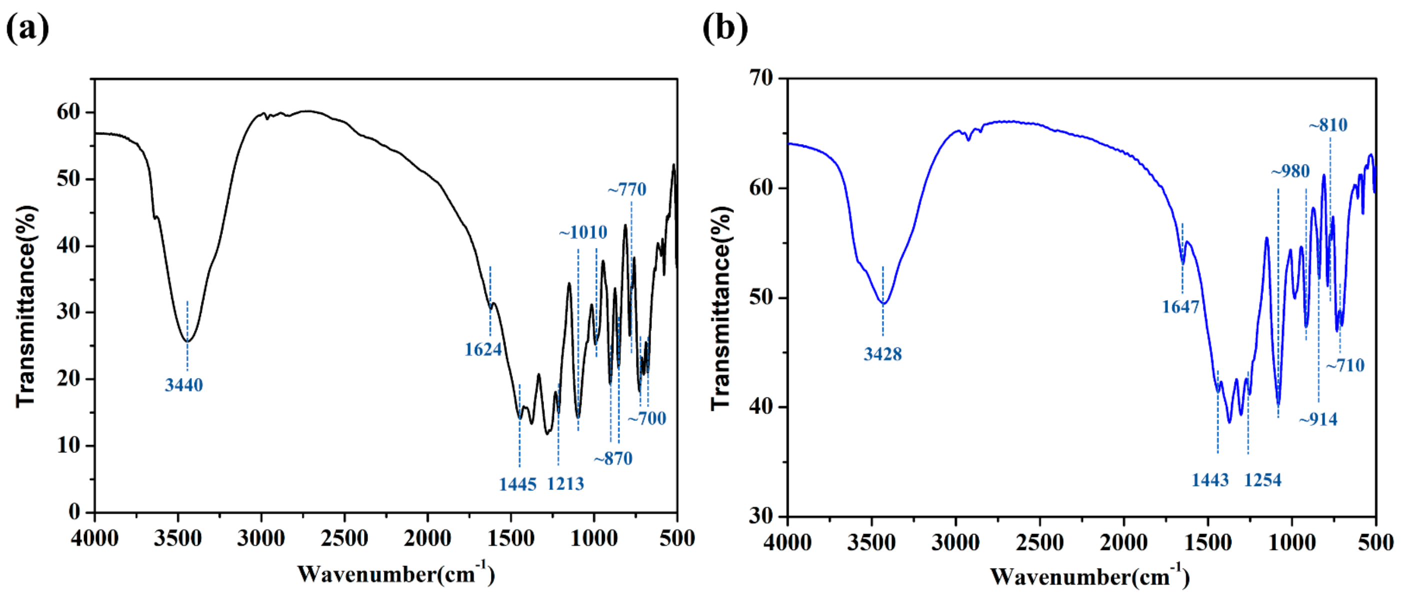

Figure S8. IR spectra of **1** (a) and **2** (b).

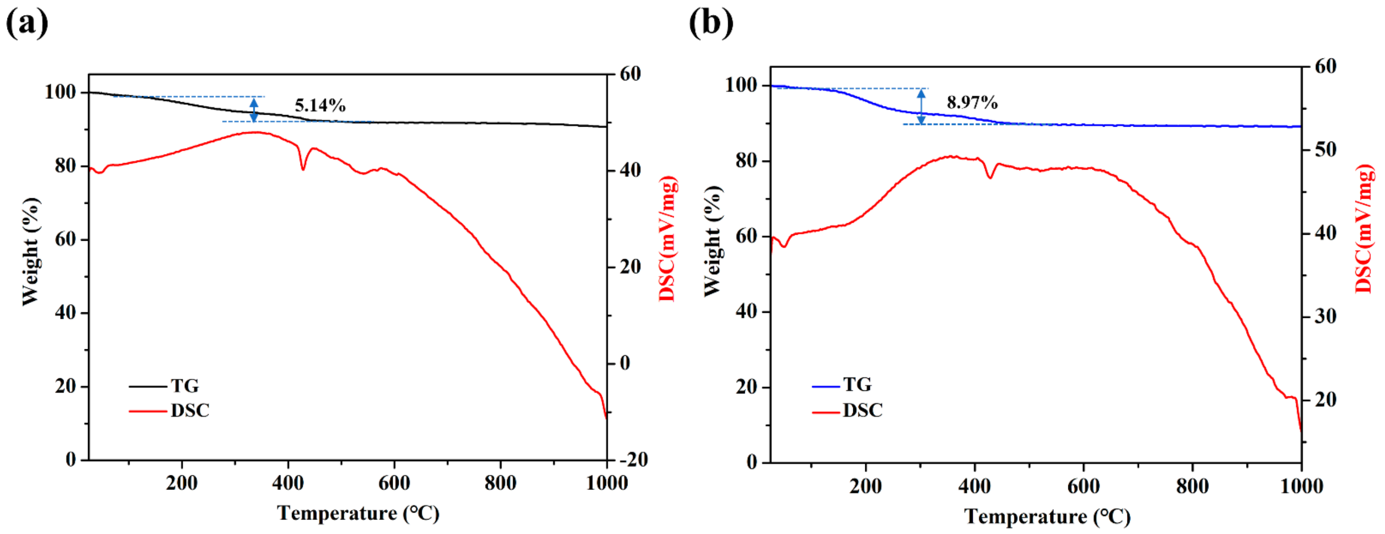

Figure S9. TG-DSC curves of **1** (a) and **2** (b).

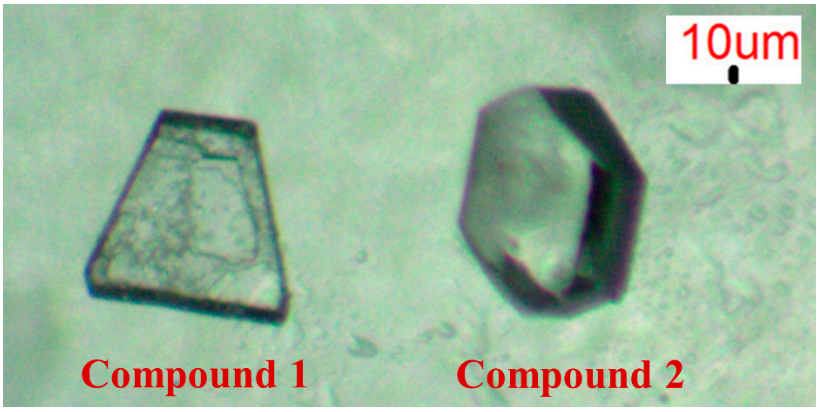

Figure S10. The morphology of compounds **1** and **2**, respectively.
